# Editorial: Growth and quality formation regulated by light in horticulture plants

**DOI:** 10.3389/fpls.2024.1414970

**Published:** 2024-05-16

**Authors:** Houcheng Liu, Jung Eek Son, Genhua Niu, Qingming Li

**Affiliations:** ^1^ College of Horticulture, South China Agricultural University, Guangzhou, China; ^2^ Department of Agriculture, Forestry and Bioresources, Seoul National University, Seoul, Republic of Korea; ^3^ Texas A&M AgriLife Research, Texas A&M University, Dallas, TX, United States; ^4^ Institute of Urban Agriculture, Chinese Academy of Agricultural Sciences, Chengdu, China

**Keywords:** light environment, growth, quality, vegetable, fruit, herb

Light plays a crucial role in the growth and metabolism of plants. It is one of the most important abiotic factors that regulate various physiological signals as well as primary and secondary metabolic responses in plants. Light intensity, spectrum, direction, photoperiod, and timing of lighting all play a role in regulating the physiological and molecular processes of plants. Light is also the most important environmental factor determining the yield and quality of horticultural crops. In this Research Topic ‘Growth and Quality Formation Regulated by Light in Horticulture Plants’, there are nine original research articles all focusing on the effects of different lighting environments on the growth and nutritional quality of fruits, vegetables, and herb.

Plants compete for sunlight and have evolved to perceive shade through both relative increases in the photon flux density of far-red (FR; 700 to 750 nm) and decreases in the flux of all photons (intensity). The FR photons and light intensity interact to control stem elongation and leaf expansion in plants. These interactions have important implications for horticultural crops. Kusuma and Bugbee studied this interaction between FR fraction and total light intensity with a range of 2 to 33% FR at 50/100, 200 and 500 µmol m^-2^ s^-1^ extended photosynthetic photon flux densities (ePPFD, 400 to 750 nm). Increasing FR light increased leaf expansion in three lettuce cultivars (rosette plant architecture) at the highest ePPFD, but not decreased expansion at the lowest ePPFD. The authors attributed this difference to biomass partitioning between leaves and stem. Increased FR favored stem elongation at low ePPFD and favored leaf expansion at high ePPFD. For cucumber seedlings (upright plant architecture), plants responded to FR differently: leaf expansion increased with increasing FR percent under all ePPFD levels showing minimal interaction. These results indicated that the interaction of FR and light intensity is species dependent.

The phytochrome photostationary state (PSS) sometimes called the phytochrome photo-equilibrium, is the ratio of active phytochrome to the total phytochrome (P_fr_/P_total_), and can be calculated from absorptivity data of isolated phytochromes (Sager et al., 1988). If the temporal PSS changes could be estimated, the effects of artificial lighting on plants could be estimated in more detail, and plant morphology and development could be controlled more efficiently and accurately. Jishi developed a model to estimate the time course of a phytochrome photostationary state (PSS) under an arbitrary light environment. The model estimated that the 90% and 99% of the PSS changes were completed using approximately 3.4 and 6.9 mmol m^-2^ of integrated end-of-day FR, respectively. The rate at which the PSS changes reached equilibrium was maximized under a red light, followed by far-red, green, and blue light. This method could be used to control phytochrome responses for horticulture via artificial lighting.

Anthocyanins not only are an important factor in promoting fruit coloration but also have a rich nutritional and medicinal values. Anthocyanin accumulation is affected by light intensity and light spectrum, especially blue light. Anthocyanins are the main pigments affecting the color and quality of purple-fruited sweet pepper (*Capsicum annuum*). Gao et al. performed the anthocyanin content determination and transcriptome analysis on pepper fruits harvested from different light treatments. The levels of delphinidin (Dp) glycosides, including Dp-3-O-rhamnoside, Dp-3-O-rutinoside, and Dp-3-O-glucoside, were highly accumulated in blue-light–treated fruit, which are mainly responsible for the appearance color of purple pepper. There were 6 structural and 12 transcription factor (TF) genes involved in the anthocyanin biosynthetic pathway. Structural gene, such as, CaUFGT as well as TFs such as CaMYC2-like and CaERF113, which were highly expressed under blue light and presented similar expression patterns consistent with Dp glycoside accumulation. These might be candidate genes for anthocyanin synthesis in response to blue-light signal.

For greenhouse crop production, the spectral quality of supplemental lighting (SL) can not only directly influence crop yield but also nutritional quality. Manipulating the spectral quality of greenhouse SL enhanced the production of secondary metabolites, which can be used for culinary, medicinal, and commercial purposes (Holopainen et al., 2018). Hammock and Sams determined the impact of supplemental blue (B) and red (R) LED lighting ratios and discrete wavelengths on flavor volatiles in hydroponically grown basil (*Ocmum basilicum* var. Italian Large leaf). They found that SL spectral quality, changes in the spectra, and daily light integral (DLI) of ambient sunlight across growing seasons, directly affected basil aroma volatile concentrations. In addition, the specific ratios of narrowband B/R wavelengths, combinations of discrete narrowband wavelengths, and broadband wavelengths directly and differentially influence the overall aroma profile as well as specific compounds. They recommend SL using B and R light at a ratio of approximately 10B/90R at 100-200 µmol. m^-2^ s^-1^ for 12-24 h. d^-1^ for sweet basil standard greenhouse production.

Sweet potato (*Ipomoea batatas* (L.) Lam) is a staple and a critical food crop in developing countries. Sweet potato leaves have been demonstrated to be more nutritious than their stems, petioles, tubers, and other vegetables. Light conditions substantially impact sweet potato leaves, which differ in their shape and metabolic profiles. Tadda et al. determined the nutritional profile of sweet potato leaves were affected by red and blue LEDs. There were higher contents of soluble protein, total phenolic compounds, flavonoids, and total antioxidant activity under blue LEDs, while higher contents of chlorophyll, soluble sugar, protein, and vitamin C under red LEDs. A total of 615 genes were differentially expressed between sweet potato leaves exposed to red and blue LEDs. Among these, 510 differentially expressed genes were upregulated in leaves grown under blue light compared with those grown under red light, while the remaining 105 genes were expressed at higher levels in the latter than in the former. Blue light significantly induced anthocyanin and carotenoid biosynthesis structural genes.

Selecting suitable light conditions according to the plant growth characteristics is one of the important approaches to cultivating high-quality vegetable seedlings. Liu et al. investigated the growth characteristics of tomato and cucumber seedlings under LED light environments (CK, B, UV-A, FR, B+UV-A, UV-A+FR, and B+FR) in plant factories with artificial light (PFALS) and the development of these seedlings after transplanting into the plastic greenhouse. The seedling height and hypocotyl length increased in treatments with far-red light supplementation, but decreased in the B treatment, in both crops. The seedling index of tomato increased in the B+UV-A treatment, while that of cucumber increased in the FR treatment. After transplanting into the plastic greenhouse, tomato plants that radiated with UV-A had greater flower numbers on the 15th day after transplanting. In cucumber plants of the FR treatment, the flowering time was significantly delayed, and the female flower exhibited at a lower node position. The light environments with UV-A and FR were more beneficial for improving the overall quality of tomato and cucumber seedlings, respectively.

Improving the light environment and enhancing the utilization of light energy by plants have become critically important in greenhouse tomato production. Artificial supplemental lighting can improve the light conditions of plant canopies. Bifacial leaves can fix more carbon than leaves with one irradiation surface when exposed to the same irradiation amount (Zhang et al., 2016). Jiang et al. assessed the transcriptomic and proteomic changes in tomato leaves under abaxial (AB) and adaxial leafy supplemental lighting (AD). Under the two methods, a total of 7352 genes and 152 proteins were differentially expressed. Significant differences were observed in genes expression levels and proteins abundances across multiple pathways, mainly including cell process, metabolism process, biological regulation, environment information processing, genetic information processing, metabolism, and organismal systems. The effect of AB on plant growth and development might be due to the increasing expression of some key genes related to plant hormone signaling, light perception, photosynthesis, plant fitness, and promoting fruit ripening. AB mainly up-regulate a series of auxin-responsive genes or factors, auxin polarity transport genes, gibberellin synthesis genes, cell cycle regulator genes, sugar transporters, and fleshy fruit ripening genes. This study provides useful knowledge for improving both the light-use efficiency of plants and fruit yield by adjusting supplemental light approaches.

Tomato is the most economically important horticultural crop. Tomato plants can be cultivated as single- or two-shoot plants. In two-shoot plants, each shoot shares the roots capacity, necessitating double the root activity or transport efficiency to sustain the same solute flux per shoot as in one-shoot plants. The combination of supplemental top-lighting with high pressure sodium lamp (HPS) and inter-lighting with LEDs increases the yield of tomato plants, with increased fruit weight being a commercially important component of this yield enhancement. The study by Paponov et al. investigated the combined effects of light and branching on fruit size and chemical fruit quality of greenhouse tomatoes. The two-shoot plants had lower yield mainly due to smaller fruit size, instead of source strength limitations, based on the evaluation of leaf weight ratio (LWR), chlorophyll index, specific leaf area (SLA), leaf dry matter percentage, and stem soluble carbohydrate accumulation. Enhanced lighting improved fruit weight and various fruit traits, such as dry matter content, total soluble carbohydrate content, and phenolic content, for both one- and two-shoot plant types. Despite lower mean fruit weight, two-shoot plants exhibited higher values for chemical fruit quality traits, indicating that the fruit growth of two-shoot plants is not limited by the available carbohydrates (source strength), but by the fruit sink strength. Overall, two-shoot branching primarily modified sink capacity, while lighting primarily affected source activity. Two-shoot cultivation reduced the xylem sap concentration of cytokinins that can inhibit the sink capacity of young fruits. Additionally, the increased hydraulic resistance associated with two-shoot plant architecture appears to improve fruit quality due to the higher solute flux from the phloem at the expense of the xylem. The stronger inhibition of sink than source activity, together with the increased hydraulic resistance in the stem, resulted in fruits that were smaller but showed higher accumulation of dry matter content and improved fruit quality traits. Notably, fruits from two-shoot plants had enhanced accumulations of dry matter and phenolic contents.

*Coptis chinensis* is a perennial, shade-tolerant, evergreen, understory medicinal plant. The rhizome (RO) of *C. chinensis*, which contains alkaloids, especially berberine, is the main effective component for its therapeutic effects. Light directly influences photosynthesis and the demand for metabolites by sinks, which indirectly regulates the redistribution of reserves (Iqbal et al., 2012). The early spring is a seasonal high-light “window” for new leaf growth and photosynthetic carbon capture by the shade-tolerant evergreen understory plants. Ke et al. conducted the study of *C. chinensis* under low and relatively high light intensities in a greenhouse. The plants grown under higher light intensity had higher starch in rhizome (RO) and higher RO biomass at the end of the year compared to those grown under lower light intensity. The photosystem II (PSII) operating efficiency [Y(II)], relative electron transport rate (rETR), and photochemical quenching (qP), as well as sucrose and glucose, in immature leaf (IL) and mature leaf (ML) under relatively higher light, was higher than those under lower light. The glucose and starch concentrations in ILs at 35 d was significantly higher than that at 15 d when plants were under 200 mmol m^-2^ s^-1^, while they were not significantly changed and remained low at 50 mmol m^-2^ s^-1^. The proportion of photosynthetic transport from ILs to MLs was significantly higher than that from MLs to ILs under the 50 mmol m^-2^ s^-1^ limit. Total P concentration in ILs was lower under relatively higher light, but there was no difference in nucleic acid P concentration in ILs under the two light intensity treatments. The alkaloid concentration in RO was lower under 200 mmol m^-2^ s^-1^ than that under 50 mmol m^-2^ s^-1^. Relatively higher light reduces the need for carbohydrates and P stored in the RO to support IL growth by (1) accelerating the sink-to-source transition in ILs, which inhibits the use of reserves in the RO; (2) using energy from MLs to support IL growth, thereby reducing RO reserve consumption, and (3) reducing the demand for P by investing less in the development of photosynthetic machinery.

In summary, the articles in this Research Topic provide a broad overview of the key roles of light environment on growth and quality of horticultural crops. This will highlight innovative and emerging areas in light regulation of horticultural crops and will inspire researchers with a wide range of research interests.

## Author contributions

HL: Writing – original draft, Writing – review & editing. JS: Writing – review & editing. GN: Writing – original draft, Writing – review & editing. QL: Writing – review & editing.
